# A palmitoylation-associated three-gene signature predicts prognosis, immune features, and drug sensitivity in gastric cancer: functional validation of CTHRC1

**DOI:** 10.3389/fimmu.2026.1893438

**Published:** 2026-09-25

**Authors:** Hongmei Sheng, Xuejun Li, Chen Zhang, Miaomiao Song, Juan Xu, Jun Ma

**Affiliations:** 1 Second Department of Geriatrics, The Second Affiliated Hospital of Anhui University of Chinese Medicine, Hefei, China; 2 Peking University Shenzhen Hospital/School of Clinical Medicine, Hubei University of Medicine, Shiyan, Hubei, China; 3 Department of Encephalopathy, The Second Affiliated Hospital of Anhui University of Chinese Medicine, Hefei, China; 4 Department of Oncology, The Fourth Affiliated Hospital of Anhui Medical University, Hefei, China

**Keywords:** drug sensitivity, gastric cancer, palmitoylation-associated genes, prognosis, tumor microenvironment

## Abstract

**Background:**

Gastric cancer (GC) is a heterogeneous malignancy that requires improved molecular tools for prognostic stratification. Protein palmitoylation regulates membrane localization and signaling, but the clinical relevance of palmitoylation-related genes (PRGs) in GC remains unclear.

**Methods:**

Gene Expression Omnibus, The Cancer Genome Atlas, and GeneCards data were integrated to identify PRGs. Differential expression analysis, weighted gene co-expression network analysis, and three machine-learning algorithms were used to select key genes. Diagnostic and prognostic performance was evaluated in TCGA-STAD, platform-specific GEO cohorts, and an institutional cohort of 80 paired tissues. Immune infiltration, single-cell RNA sequencing, immunotherapy-related indices, and GDSC-based drug sensitivity were analyzed. CTHRC1 was further examined using clinical specimens, cell assays, and xenografts.

**Results:**

Thirteen differentially expressed PRGs were identified, and SERPINE1, CTHRC1, and EGR1 were selected. The integrated diagnostic model achieved AUCs of 0.935 in TCGA-STAD, 1.000 in GSE54129, and 0.916 in GSE118916, respectively. The three-gene score stratified overall survival in the total, training, and testing TCGA-STAD cohorts and in GSE84437, GSE236522, and the institutional cohort. It remained independently associated with survival in multivariable analysis. High-risk tumors showed increased stromal and immune scores, altered immune-cell composition, higher checkpoint expression, and higher TIDE scores. Single-cell analysis localized SERPINE1 and CTHRC1 mainly to fibroblast-related compartments. CTHRC1 knockdown reduced GC-cell proliferation, clonogenicity, migration, invasion, and xenograft growth while increasing apoptosis.

**Conclusion:**

The SERPINE1/CTHRC1/EGR1 signature provides prognostic and immune-stromal information in GC, and the experiments support a tumor-promoting role for CTHRC1. The findings define a palmitoylation-associated transcriptional framework but do not establish a palmitoylation-dependent mechanism.

## 1 Introduction

Gastric cancer (GC) remains one of the leading causes of cancer-related mortality worldwide, with marked geographic variation and a particularly high burden in East Asia (1). Despite improvements in endoscopic detection, surgical techniques, perioperative chemotherapy, targeted therapy, and immune checkpoint blockade (2–4), outcomes remain poor for many patients with advanced or recurrent disease (5). Current treatment decisions still rely heavily on anatomic staging and histopathological assessment, but patients with similar tumor-node-metastasis (TNM) stage can show distinct clinical courses and therapeutic responses (6). Additional molecular and microenvironmental biomarkers are therefore required to refine prognostic assessment and treatment research.

Large-scale genomic and transcriptomic studies have changed the biological understanding of GC. The Cancer Genome Atlas (TCGA) classified gastric adenocarcinoma into Epstein-Barr virus-positive, microsatellite instability, genomically stable, and chromosomal instability subtypes, each showing distinct molecular and clinical features (7). The Asian Cancer Research Group further identified expression-based molecular subtypes with different recurrence patterns and survival outcomes (8). These frameworks demonstrate that GC is not a single disease entity but a heterogeneous group of tumors shaped by tumor-intrinsic alterations and microenvironmental states. However, molecular classification has not fully solved the need for simple and reproducible prognostic tools that can be linked to biological mechanisms.

GC heterogeneity reflects multiple cancer hallmark programs, including sustained proliferative signaling, invasion, immune evasion, and microenvironmental remodeling (9). Protein palmitoylation, also known as S-acylation, is a reversible post-translational lipid modification in which fatty acyl chains are attached to cysteine residues (10). This modification controls protein trafficking, membrane association, subcellular localization, and signal transduction, thereby influencing receptor signaling, cell adhesion, and migration (11). Cancer-focused studies have shown that dysregulated palmitoylation can modulate oncogenic signaling, metastatic behavior, and tumor-immune interactions (12, 13). Curated resources and enzymology studies have also improved the annotation of palmitoylated proteins and the specificity of palmitoyltransferases, providing a basis for systematic analysis of palmitoylation-related genes (PRGs) (14, 15).

The tumor microenvironment (TME) is increasingly recognized as a determinant of GC progression and treatment resistance. Tumor cells interact with cancer-associated fibroblasts (CAFs), tumor-associated macrophages (TAMs), endothelial cells, regulatory T cells and extracellular matrix components to create microenvironments that support invasion, angiogenesis, immune escape and drug resistance (16). Therapeutic targeting of the TME has become an important strategy because stromal and immune barriers can limit the efficacy of cytotoxic therapy and immune checkpoint blockade (17). CAFs contribute to matrix remodeling, growth factor production and immune exclusion (18), whereas TAMs can promote suppressive myeloid programs and attenuate antitumor immunity (19). These observations make it important to determine whether PRG-associated molecular features reflect not only tumor cell states but also TME organization.

These observations suggest that PRG-associated transcriptomic patterns may provide a biologically interpretable entry point for GC stratification. However, most existing GC biomarker studies focus on conventional oncogenic pathways or immune checkpoints, whereas the relationship among PRGs, prognosis, immune-stromal remodeling, single-cell localization and functional validation remains insufficiently defined. A model that integrates public omics data with clinical samples and experimental assays may therefore offer a more credible framework for identifying actionable PRG-related biomarkers.

In addition to prognosis and immunotherapy response, drug sensitivity represents another critical dimension of precision oncology. GC treatment still relies on fluoropyrimidine-, platinum-, taxane-, and irinotecan-based regimens, but therapeutic responses vary markedly across patients. Pharmacogenomic resources such as the Genomics of Drug Sensitivity in Cancer (GDSC) provide a useful framework for linking molecular features to drug-response patterns in cancer cell lines (20). Therefore, integrating PRG-associated molecular stratification with drug-sensitivity analysis may provide additional clues for individualized therapeutic selection.

In this study, we integrated GEO, TCGA, GeneCards, single-cell, and pharmacogenomic datasets to identify PRG-associated biomarkers in GC. Differential expression analysis, WGCNA, and machine learning were combined to screen key DEPRGs; diagnostic and prognostic models were then evaluated across public and institutional cohorts. We further characterized immune-stromal features, assessed exploratory immunotherapy-related signals, and examined GDSC-based drug sensitivity patterns. Clinical validation and functional experiments were used to determine whether CTHRC1 contributes to GC progression. Because no direct palmitoylation assay was performed, the study was designed to evaluate a transcriptional signature associated with a palmitoylation-related gene set rather than to establish a palmitoylation-dependent mechanism. The overall analytical workflow is summarized in **Figure 1**.

**Figure 1:**
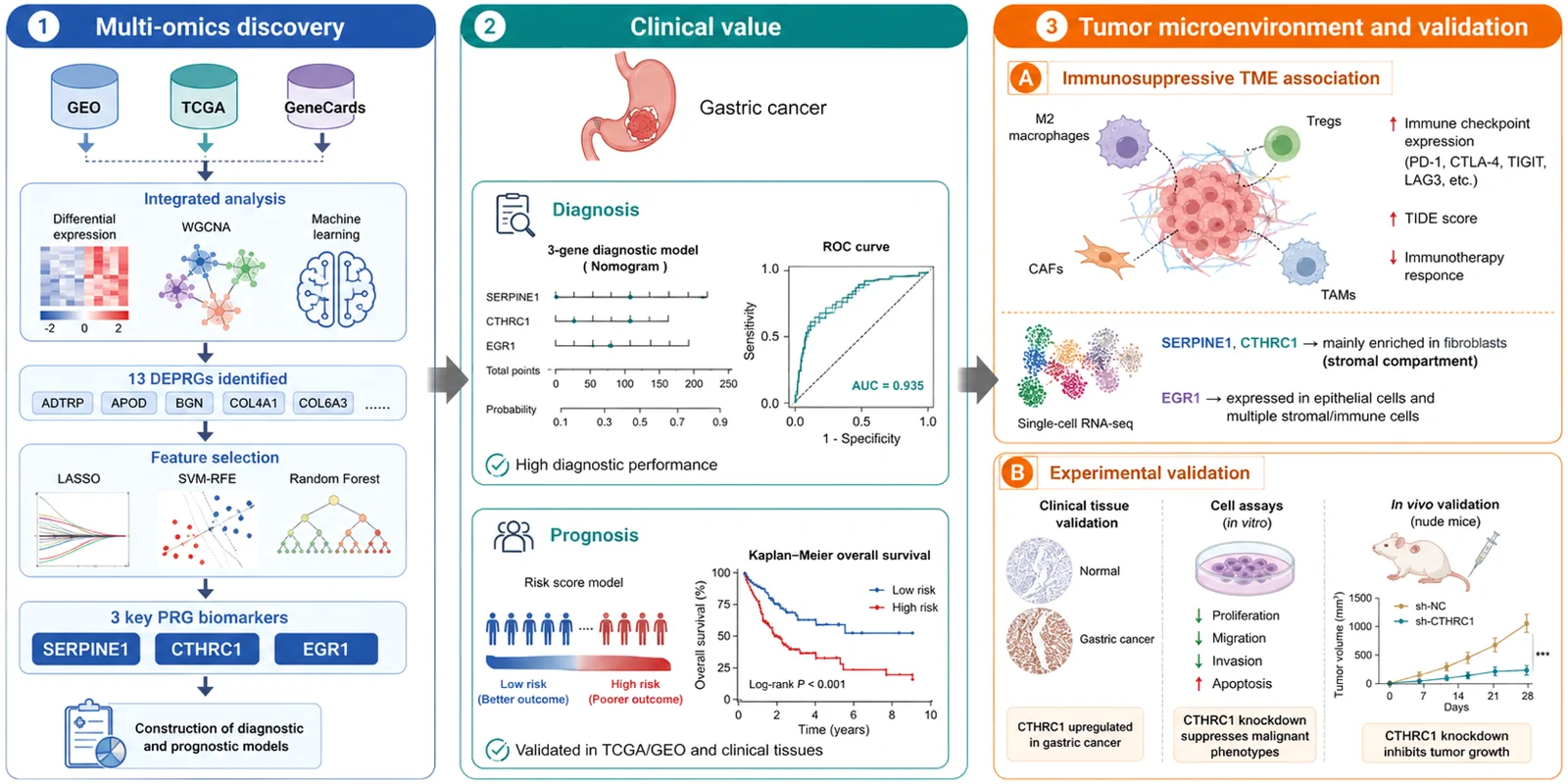
Schematic overview of the multi-omics strategy used to identify palmitoylation-related gene (PRG)-associated biomarkers and evaluate prognostic, immune-stromal, pharmacogenomic, and experimental features in gastric cancer (GC). The embedded survival and experimental elements are schematic representations of the workflow; quantitative results are presented in the corresponding figures.

## 2 Materials and methods

### 2.1 Data acquisition and preprocessing

Gene expression profiles and clinical information were retrieved from the Gene Expression Omnibus (GEO) database (21). GSE54129 and GSE118916 were used for the initial differential-expression and co-expression analyses. For each dataset, the normalized matrix supplied by the original study was log2-transformed when required, probes were mapped to official gene symbols, and duplicate probes were summarized by their mean expression. The two datasets were then restricted to common genes, merged, and adjusted for study-specific batch effects using the ComBat function in the sva package. Expression distributions, sample clustering, and principal-component patterns were examined before and after correction to assess the effectiveness of batch correction and the preservation of biological separation between control and tumor samples. RNA sequencing, somatic-mutation, and clinicopathological data from the TCGA stomach adenocarcinoma cohort (TCGA-STAD) were obtained from The Cancer Genome Atlas (TCGA) (22) and used for diagnostic-model fitting and prognostic analyses; cases without complete overall-survival information were excluded from survival modeling. GSE54129 and GSE118916 were additionally examined as platform-specific diagnostic evaluation cohorts, while GSE84437 and GSE236522 were used for external prognostic evaluation. Because the GEO cohorts contributed to gene discovery, their diagnostic results are described as cross-dataset/platform evaluations rather than completely independent feature-discovery validation. Palmitoylation-related genes (PRGs) were retrieved from GeneCards (version 6.1; https://www.genecards.org/) using the search term “palmitoylation.” All genes with a GeneCards Relevance score >1 were retained, yielding 3,307 PRGs without manual addition or exclusion. This prespecified cutoff excluded weakly linked entries while preserving broad pathway coverage; the complete gene list and scores are provided in **Supplementary Table 1** (23). TCGA-STAD clinical variables are summarized in **Supplementary Table 2**.

### 2.2 Differential expression and functional enrichment analysis

Differential expression analysis between GC and control tissues was performed using limma (24). Genes with |log2 fold change| >1 and Benjamini–Hochberg-adjusted P < 0.05 were defined as differentially expressed genes (DEGs). Heatmaps and volcano plots were used to visualize expression patterns. Gene Ontology (GO) and Kyoto Encyclopedia of Genes and Genomes (KEGG) enrichment analyses were performed using clusterProfiler (25), and gene set enrichment analysis (GSEA) was used to assess coordinated pathway-level differences (26).

### 2.3 Weighted gene co-expression network analysis

WGCNA was used to identify co-expression modules associated with GC status (27). Samples and genes were screened for missingness and outliers before network construction. The pickSoftThreshold function was used to select a power approximating scale-free topology. An adjacency matrix and topological overlap matrix were constructed, genes were clustered by dynamic tree cutting, and module–trait relationships were calculated. The blue module was selected because it showed the strongest significant positive association with GC status.

### 2.4 Identification and characterization of differentially expressed palmitoylation-related genes

Differentially expressed palmitoylation-related genes (DEPRGs) were defined as the intersection of DEGs, genes in the GC-associated WGCNA module, and GeneCards-derived PRGs. Expression differences between GC and control tissues were assessed using Wilcoxon rank-sum tests. Univariate Cox regression was used to screen OS-associated DEPRGs. Spearman correlation analysis was used to examine relationships among DEPRGs, and genomic localization was visualized using circos and coordinate plots.

### 2.5 Machine-learning screening of key PRG biomarkers

Three complementary machine-learning methods were applied to the 13 DEPRGs. Least absolute shrinkage and selection operator (LASSO) logistic regression was implemented using glmnet with 10-fold cross-validation, and the penalty parameter was selected at the minimum cross-validated error (28). Support vector machine-recursive feature elimination (SVM-RFE) used 10-fold cross-validation to compare feature subsets and selected the subset with the lowest cross-validated classification error. Random-forest analysis used 500 trees and ranked variables according to model-based importance (29). Genes retained by all three algorithms were defined as robust candidate biomarkers. Feature selection and coefficient estimation were completed without using GSE84437, GSE236522, or the institutional validation cohorts. The TCGA prognostic cohort was subsequently divided at approximately 1:1 into training and testing subsets, thereby separating model development from internal assessment and reducing information leakage.

### 2.6 Diagnostic model construction and validation

A logistic-regression diagnostic model incorporating SERPINE1, CTHRC1, and EGR1 was fitted in TCGA-STAD. Diagnostic performance of each gene and the integrated model was evaluated by receiver operating characteristic (ROC) analysis and the corresponding area under the curve (AUC) using pROC (30). Calibration curves assessed agreement between predicted and observed disease probability, and decision-curve analysis estimated net clinical benefit (31). GSE54129 and GSE118916 were evaluated separately to examine the reproducibility of diagnostic discrimination across independent expression platforms. Because these GEO datasets also contributed to the upstream discovery screen, their results were not interpreted as fully independent feature-selection validation.

### 2.7 Prognostic risk-score model and survival analysis

A PRG-associated prognostic score was constructed by multivariable Cox regression (32): risk score = 0.1785 × SERPINE1 + 0.0706 × CTHRC1 + 0.0098 × EGR1. The evaluable TCGA-STAD cases were randomly divided at approximately 1:1 into training and testing subsets for internal validation. Within the total, training, and testing cohorts, patients were divided into high- and low-risk groups using the median score. GSE84437 and GSE236522 were evaluated independently using cohort-specific cutoffs because expression scales differ between RNA sequencing and microarray platforms. Kaplan–Meier curves and log-rank tests compared overall survival (OS), Cox models estimated hazard ratios (HRs) and 95% confidence intervals (CIs), and time-dependent receiver operating characteristic (ROC) curves evaluated 1-, 3-, and 5-year performance. The external cohorts were not used to estimate model coefficients.

### 2.8 Mutation, tumor mutational burden, and molecular subtype analyses

Somatic mutation data from TCGA-STAD were analyzed using maftools (33). Oncoplots, variant-classification summaries, single-nucleotide variant distributions, and lollipop plots were generated for the core PRGs. Tumor mutational burden (TMB) was calculated as the number of somatic mutations per megabase. Consensus clustering was performed using ConsensusClusterPlus (34). Principal component analysis (PCA) was used to visualize subtype separation, and gene set variation analysis (GSVA) was applied to compare pathway activity between molecular subtypes (35).

### 2.9 Immune infiltration and TME score analysis

Immune infiltration was estimated using complementary algorithms. Estimation of STromal and Immune cells in MAlignant Tumor tissues using Expression data (ESTIMATE) was used to infer StromalScore, ImmuneScore, and ESTIMATEScore (36). CIBERSORT was used to estimate the relative abundance of 22 immune-cell populations (37). xCell and quanTIseq were used as additional robustness checks (38, 39). The Tumor Immune Estimation Resource (TIMER) and Estimating the Proportions of Immune and Cancer cells (EPIC) were also applied as orthogonal deconvolution approaches (40, 41). Spearman correlation analysis was used to evaluate associations between the risk score and immune infiltration.

### 2.10 Single-cell RNA sequencing analysis

Single-cell RNA sequencing data from STAD_GSE167297 were obtained from the Tumor Immune Single-cell Hub (TISCH) resource (42). Uniform manifold approximation and projection (UMAP) plots were used to visualize cell clusters and major cell lineages. Cell annotations were based on canonical marker genes. Expression patterns of SERPINE1, CTHRC1, and EGR1 were displayed using violin plots and feature plots. Spearman correlation analysis was used to evaluate associations between core PRGs and cancer-associated fibroblast (CAF) or tumor-associated macrophage (TAM) marker genes.

### 2.11 Immune checkpoint, tumor immune dysfunction and exclusion, and immunotherapy response analysis

Immune checkpoint expression was compared between the high-risk and low-risk groups. Spearman correlation analysis was used to evaluate associations between immune checkpoint expression and the risk score. Tumor Immune Dysfunction and Exclusion (TIDE) was used to infer T-cell dysfunction, immune exclusion, and predicted checkpoint-blockade response (43). IMvigor210, a urothelial-cancer cohort treated with an antibody against programmed death-ligand 1 (PD-L1), was included only as exploratory cross-cancer evidence and was not considered a GC validation cohort. An institutional GC cohort with recorded immunotherapy response was used for additional exploratory assessment. These analyses were interpreted as predictive associations rather than substitutes for prospective treatment-response validation.

### 2.12 GDSC-based drug sensitivity analysis

Drug sensitivity was analyzed using the Genomics of Drug Sensitivity in Cancer (GDSC) resource (20). GC cell lines with matched expression and response data were assigned a cell-line risk score using the established three-gene formula and divided at the median. Drug response was represented by the natural logarithm-transformed half-maximal inhibitory concentration [ln(IC50)]; lower ln(IC50) indicated greater *in vitro* sensitivity. Differences between the high-risk and low-risk groups were tested using the Wilcoxon rank-sum test. The analysis was restricted to representative chemotherapeutic and targeted agents and was considered hypothesis-generating because cell-line pharmacogenomics does not reproduce patient-level tumor heterogeneity, stromal interactions, pharmacokinetics, or immune effects.

### 2.13 Drug/compound-gene interaction analysis

Potential drug/compound–gene associations for SERPINE1, CTHRC1, and EGR1 were retrieved from the Drug–Gene Interaction Database (DGIdb; https://www.dgidb.org/) (44) and visualized in Cytoscape (45). Database edges were interpreted as reported molecular or pharmacological associations, not as evidence of efficacy in GC. The network was therefore used only to prioritize hypotheses for subsequent experimental testing.

### 2.14 Clinical sample collection, processing, and ethics approval

Eighty paired GC and adjacent non-tumor tissues were obtained from the Fourth Affiliated Hospital of Anhui Medical University. All patients had pathologically confirmed GC. Fresh specimens used for quantitative reverse-transcription polymerase chain reaction (qRT-PCR) were stored in RNAlater (Thermo Fisher Scientific, AM7021) or snap-frozen in liquid nitrogen immediately after resection. Formalin-fixed paraffin-embedded specimens were fixed in 10% neutral buffered formalin (Servicebio, G1101, Wuhan, China) and processed using standard histological procedures. For clinical-tissue immunohistochemistry (IHC), deparaffinized sections underwent antigen retrieval and blocking, followed by incubation with primary antibodies against SERPINE1, CTHRC1, or EGR1, horseradish peroxidase (HRP)-conjugated secondary antibodies, 3,3′-diaminobenzidine (DAB) development, and hematoxylin counterstaining. Representative fields were acquired under the same imaging conditions. Follow-up information was collected for survival analysis. The protocol was approved by the Ethics Committee of the Fourth Affiliated Hospital of Anhui Medical University (KYXM-202509-006), and written informed consent was obtained from all participants.

### 2.15 Cell culture, CTHRC1 knockdown and molecular validation

The human GC cell lines AGS (American Type Culture Collection [ATCC], CRL-1739), HGC-27 (Procell, CL-0107), and MKN-45 (Procell, CL-0292), together with the normal gastric epithelial cell line GES-1 (Procell, CL-0563), were used for expression validation and functional assays. AGS cells were cultured in F-12K medium (ATCC, 30-2004) supplemented with 10% fetal bovine serum (FBS; ATCC, 30-2020) and 1% penicillin-streptomycin (Gibco, 15140122). HGC-27, MKN-45, and GES-1 cells were cultured in RPMI-1640 medium (Procell, PM150110) containing 10% FBS (Procell, 164210) and 1% penicillin-streptomycin solution (Procell, PB180120). All cells were maintained at 37 °C in a humidified incubator with 5% CO_2_ and were routinely tested for mycoplasma contamination.

CTHRC1 knockdown was achieved using short hairpin RNA (shRNA) lentiviral vectors synthesized by GeneChem (Shanghai, China), with a non-targeting shRNA as the negative control (sh-NC). Viral transduction was performed according to the manufacturer’s instructions, and stable cells were selected with puromycin (Beyotime, ST551). Three independent CTHRC1-targeting constructs were initially screened by qRT-PCR and Western blotting; the construct showing the most stable and efficient knockdown was selected for the subsequent functional experiments.

Total ribonucleic acid (RNA) was extracted from cells and tissues using TRIzol reagent (Invitrogen, 15596026). RNA concentration and purity were determined using a NanoDrop spectrophotometer. Complementary DNA (cDNA) was synthesized using PrimeScript RT Reagent Kit with genomic DNA (gDNA) Eraser (Takara, RR047A), and qRT-PCR was performed using TB Green Premix Ex Taq II (Takara, RR820A) on a real-time PCR system. Primers were synthesized by Sangon Biotech (Shanghai, China), and glyceraldehyde-3-phosphate dehydrogenase (GAPDH) was used as the internal control. Relative expression levels were calculated using the 2^−ΔΔCt^ method in accordance with the Minimum Information for Publication of Quantitative Real-Time PCR Experiments (MIQE) guidelines (46).

For Western blotting, total protein was extracted using radioimmunoprecipitation assay (RIPA) lysis buffer (Beyotime, P0013B) supplemented with phenylmethylsulfonyl fluoride (PMSF; Beyotime, ST506). Protein concentration was quantified using a bicinchoninic acid (BCA) Protein Assay Kit (Beyotime, P0012). Equal amounts of protein were separated by sodium dodecyl sulfate-polyacrylamide gel electrophoresis (SDS-PAGE) and transferred to polyvinylidene difluoride (PVDF) membranes (Millipore, IPVH00010). Membranes were blocked with 5% non-fat milk and incubated overnight at 4 °C with primary antibodies against CTHRC1 (Proteintech, 16534-1-AP) and GAPDH (Proteintech, 60004-1-Ig). HRP-conjugated goat anti-rabbit immunoglobulin G (IgG; Proteintech, SA00001-2) or goat anti-mouse IgG (Proteintech, SA00001-1) was used as the secondary antibody. Signals were visualized using an enhanced chemiluminescence reagent (Beyotime, P0018S).

### 2.16 *In vitro* functional assays

Cell Counting Kit-8 (CCK-8) assays were performed to evaluate cell proliferation after CTHRC1 knockdown. Briefly, cells were seeded into 96-well plates and cultured for 0, 24, 48, and 72 h. CCK-8 reagent (Dojindo, CK04) was added to each well and incubated according to the manufacturer’s protocol. Absorbance was measured at 450 nm using a microplate reader.

DNA synthesis was assessed using a BeyoClick 5-ethynyl-2′-deoxyuridine (EdU)-594 Cell Proliferation Kit (Beyotime, C0078S). Cells were incubated with EdU working solution, fixed with 4% paraformaldehyde (Beyotime, P0099), permeabilized, reacted with click additive solution, and counterstained with 4′,6-diamidino-2-phenylindole (DAPI; Beyotime, C1002). Images were captured under a fluorescence microscope, and EdU-positive cells were quantified from randomly selected fields.

For colony formation assays, control and CTHRC1-knockdown cells were seeded into six-well plates at low density and cultured for 10–14 days. Colonies were fixed with 4% paraformaldehyde and stained with crystal violet staining solution (Beyotime, C0121). Colonies containing more than 50 cells were counted.

Wound-healing assays were performed under low-serum conditions to reduce proliferation-related confounding. Cells were seeded into six-well plates and grown to near confluence. A linear wound was generated using a sterile 200-µL pipette tip, and detached cells were removed with phosphate-buffered saline (PBS; Gibco, 10010023). Cells were then cultured in serum-free or 1% FBS medium. Images were acquired at 0 and 24 h, and migration rates were quantified using ImageJ software (47).

Transwell assays were used to evaluate migration and invasion. For migration assays, cells were seeded into the upper chamber of 24-well Transwell inserts with 8-µm pores (Corning, 3422) in serum-free medium, while complete medium containing 10% FBS was added to the lower chamber. For invasion assays, the upper chambers were pre-coated with Matrigel Basement Membrane Matrix (Corning, 354234). After incubation, cells on the lower membrane surface were fixed with 4% paraformaldehyde, stained with crystal violet, imaged under a microscope, and counted in randomly selected fields.

### 2.17 Xenograft model and histological analysis

Stable negative-control shRNA (sh-NC) and CTHRC1-targeting shRNA (sh-CTHRC1) HGC-27 cells were injected subcutaneously into BALB/c nude mice purchased from Beijing Vital River Laboratory Animal Technology Co., Ltd. Five mice were included in each group. Tumor volume was measured serially and calculated as length × width²/2. At the endpoint, tumors were excised, weighed, and fixed in 10% neutral buffered formalin (Servicebio, G1101). All animal procedures complied with institutional animal-care guidelines and were approved by the Experimental Animal Welfare Ethics Committee of Peking University Shenzhen Hospital (No. 2025-1140). Apoptosis was assessed by terminal deoxynucleotidyl transferase dUTP nick-end labeling (TUNEL) using an *In Situ* Cell Death Detection Kit, POD (Roche, 11684817910), according to the manufacturer’s instructions. For IHC, sections were incubated with antibodies against CTHRC1 (Proteintech, 16534-1-AP), Bax (Abcam, ab32503), cleaved caspase-3 (Abcam, ab32042), and Bcl-2 (Abcam, ab182858), followed by HRP-conjugated secondary antibodies and DAB development. Histological quantification was performed using ImageJ (47).

### 2.18 Statistical analysis and software

Unless otherwise specified, quantitative *in vitro* experiments were performed with three independent biological replicates, and data are presented as the mean ± standard deviation (SD). Paired tissue comparisons were performed using the paired Wilcoxon signed-rank test. Two independent groups were compared using a two-sided Student’s t-test for approximately normally distributed data or the Wilcoxon rank-sum test for non-normally distributed data. Multiple-group comparisons were performed using one-way analysis of variance (ANOVA) followed by Dunnett’s or Tukey’s multiple-comparison test, as appropriate. CCK-8 and tumor-growth curves were analyzed using two-way ANOVA with multiple-comparison correction. Survival differences were evaluated using the log-rank test, and Cox proportional-hazards models were used to estimate HRs and 95% CIs. Spearman’s correlation coefficient was used for correlation analyses. The Benjamini–Hochberg method was applied to control the false-discovery rate in multiple transcriptomic comparisons. All statistical tests were two-sided, and P < 0.05 was considered statistically significant.

All bioinformatics and statistical analyses were performed using R version 4.4.2. The R packages and corresponding versions used in this study were limma 3.62.2, sva 3.54.0, WGCNA 1.73, clusterProfiler 4.14.6, org.Hs.eg.db 3.20.0, glmnet 4.1-8, randomForest 4.7-1.2, e1071 1.7-16, caret 6.0-94, pROC 1.18.5, rms 6.8-2, survival 3.8-3, survminer 0.5.0, timeROC 0.4, maftools 2.22.0, ConsensusClusterPlus 1.70.0, GSVA 2.0.7, and ggplot2 3.5.1.

## 3 Results

### 3.1 Differential expression and functional enrichment analysis

GSE54129 and GSE118916 were normalized within their original platforms, mapped to common gene symbols, merged, and adjusted for dataset-specific effects. In the corrected expression matrix, the project annotation remained visible, but the dominant separation in the heatmap was between control and tumor tissues rather than between the two GEO studies (**Figure 2A**). This pattern indicated that the merged dataset retained the biological contrast required for downstream screening. Differential-expression analysis identified a broad set of upregulated and downregulated transcripts, as illustrated by the symmetrical distribution of significant genes in the volcano plot (**Figure 2B**).

**Figure 2:**
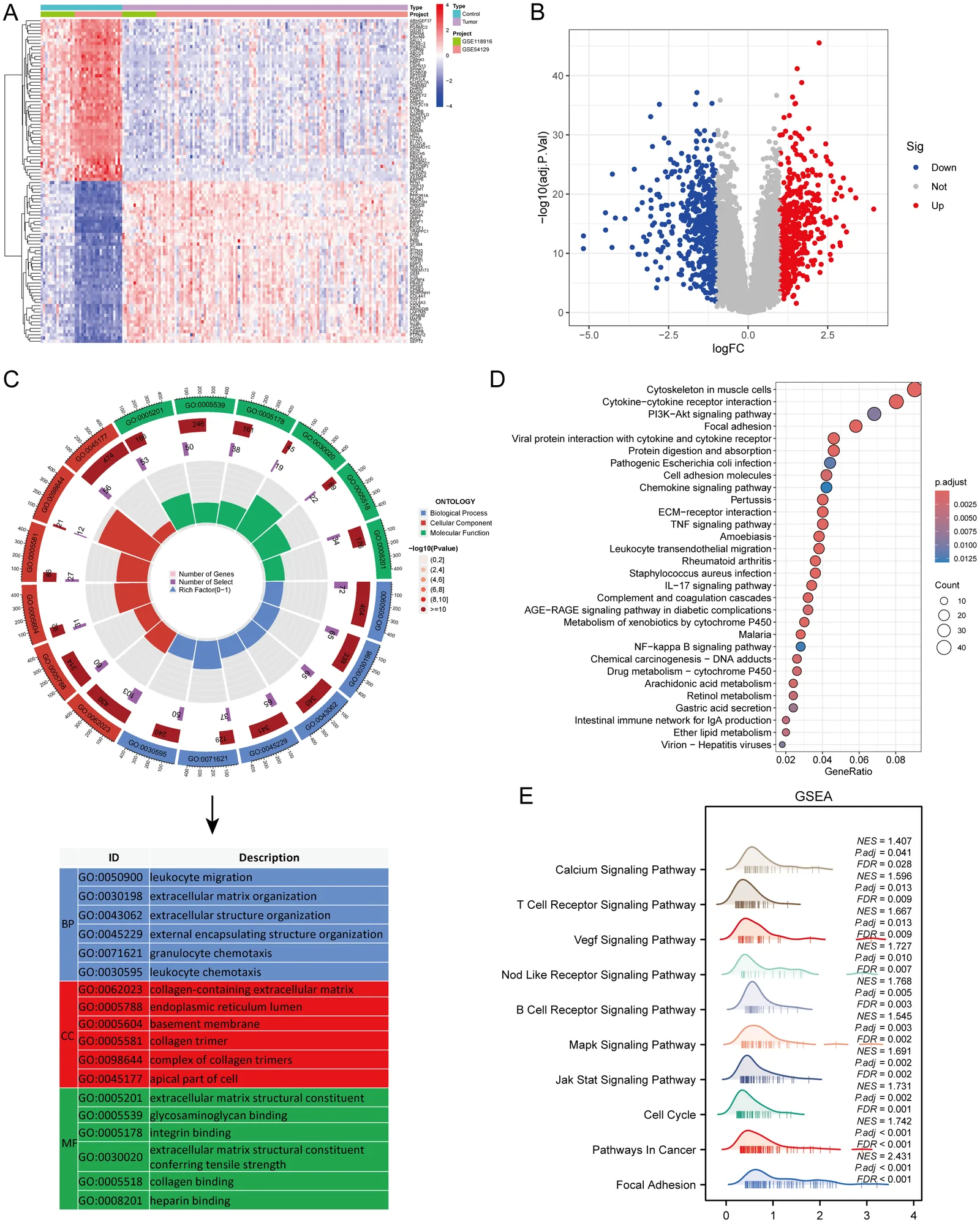
Differential expression and functional enrichment in gastric cancer (GC). GSE54129 and GSE118916 were normalized separately, merged by common gene symbols, and adjusted for dataset-specific batch effects before analysis. **(A)** Heatmap of differentially expressed genes (DEGs), with sample type labeled as control or tumor and dataset of origin labeled as GSE54129 or GSE118916. **(B)** Volcano plot of upregulated and downregulated DEGs. **(C)** Gene Ontology (GO) enrichment analysis. **(D)** Kyoto Encyclopedia of Genes and Genomes (KEGG) enrichment analysis. **(E)** Gene set enrichment analysis (GSEA) of representative pathways. Differential-expression P values were adjusted using the Benjamini–Hochberg method.

Gene Ontology (GO) enrichment indicated that the differentially expressed genes were concentrated in biological processes involving leukocyte migration, granulocyte chemotaxis, extracellular-matrix organization, and extracellular-structure organization. The enriched cellular-component terms included collagen-containing extracellular matrix, basement membrane, endoplasmic-reticulum lumen, and collagen trimers, whereas the molecular-function terms emphasized extracellular-matrix structural constituents, glycosaminoglycan binding, integrin binding, collagen binding, and heparin binding (**Figure 2C**). These results suggested that the transcriptional difference between GC and control tissues was strongly related to matrix remodeling and immune-cell trafficking.

Kyoto Encyclopedia of Genes and Genomes (KEGG) analysis further identified cytoskeletal regulation, cytokine-cytokine receptor interaction, phosphoinositide 3-kinase (PI3K)-Akt signaling, focal adhesion, extracellular matrix (ECM)-receptor interaction, tumor necrosis factor (TNF) signaling, nuclear factor kappa B (NF-κB) signaling, interleukin-17 (IL-17) signaling, chemokine signaling, and cell-adhesion pathways (**Figure 2D**). Gene set enrichment analysis (GSEA) showed coordinated enrichment of calcium, T-cell-receptor, vascular endothelial growth factor (VEGF), nucleotide-binding oligomerization domain (NOD)-like-receptor, B-cell-receptor, mitogen-activated protein kinase (MAPK), Janus kinase-signal transducer and activator of transcription (JAK-STAT), cell-cycle, pathways-in-cancer, and focal-adhesion gene sets (**Figure 2E**). Collectively, the enrichment results connected the GC transcriptome with oncogenic signaling, inflammatory activation, vascular regulation, and stromal reorganization and therefore provided a biological basis for subsequent PRG-focused analysis.

### 3.2 Identification and diagnostic evaluation of key PRG-related genes using WGCNA and machine-learning approaches

WGCNA was used to identify coordinated expression modules associated with GC status. The selected soft-thresholding power produced an approximately scale-free network while retaining sufficient mean connectivity (**Supplementary Figure 1A**). Module-trait analysis showed that the blue module had the strongest significant positive association with the tumor phenotype (**Supplementary Figure 1B**). Dynamic tree cutting resolved multiple co-expression modules (**Supplementary Figure 1C**), and genes with high module membership in the blue module also tended to show high gene significance for GC status (**Supplementary Figure 1D**), supporting the biological relevance of this module.

Intersection of the blue-module genes with the differentially expressed genes and the 3,307 GeneCards-derived PRGs yielded 13 DEPRGs: ADTRP, APOD, BGN, COL4A1, COL6A3, CTHRC1, EGR1, FLNA, FN1, PLA2G2A, SERPINE1, SERPINF1, and TAGLN (**Figure 3A**). Most of these genes were more highly expressed in tumor tissues than in controls (**Figure 3B**). Univariate Cox analysis identified APOD, BGN, COL4A1, CTHRC1, FN1, SERPINE1, and SERPINF1 as genes significantly associated with OS (**Figure 3C**). Correlation analysis demonstrated predominantly positive relationships among the 13 DEPRGs (**Figure 3D**), whereas chromosomal and genomic localization analyses showed that these genes were distributed across multiple chromosomes (**Figures 3E, F**). Immune-cell correlations associated with the 13-gene set are shown in **Supplementary Figure 2**.

**Figure 3:**
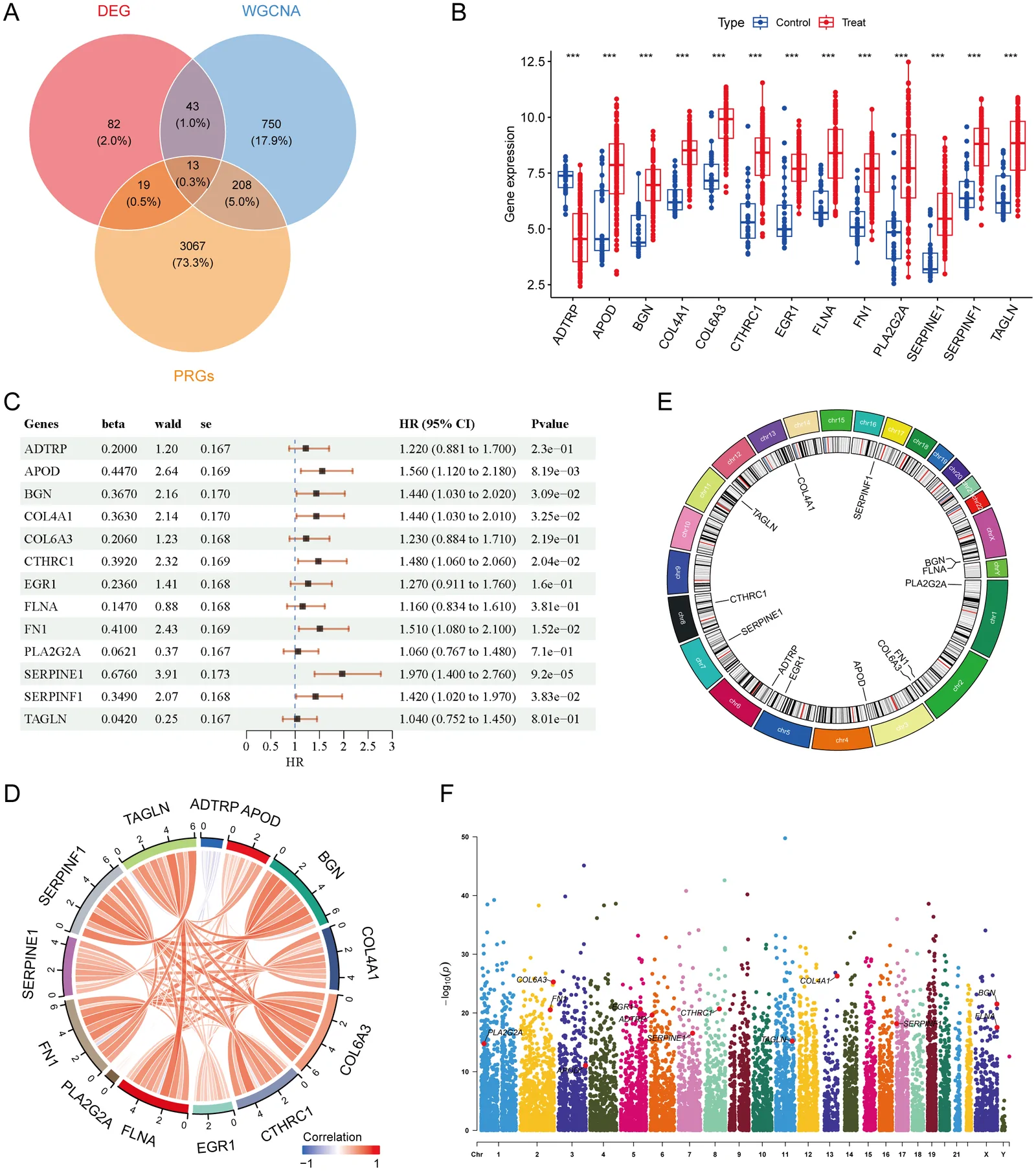
Identification and characterization of differentially expressed palmitoylation-related genes (DEPRGs) in gastric cancer. **(A)** Intersection of differentially expressed genes, genes in the GC-associated WGCNA module, and GeneCards-derived palmitoylation-related genes. **(B)** Expression differences of the 13 DEPRGs between control and tumor tissues. **(C)** Univariate Cox regression analysis of overall survival. **(D)** Correlation network among the 13 DEPRGs. **(E, F)** Chromosomal and genomic localization of the DEPRGs. ***P < 0.001.

Three complementary machine-learning methods were then applied to reduce feature-selection dependence on a single algorithm. LASSO progressively reduced the coefficients as the penalty increased and selected the minimum-error solution by 10-fold cross-validation (**Figures 4A, B**). SVM-RFE achieved its highest cross-validated accuracy with 11 features (accuracy = 0.95) and its lowest cross-validated error at the same feature number (error = 0.0496; **Figures 4C, D**). Random-forest error stabilized across 500 trees, and the importance ranking identified COL6A3 and SERPINE1 as the highest-ranked variables, with CTHRC1 and EGR1 also among the leading features (**Figures 4E, F**). The intersection of the LASSO, SVM-RFE, and random-forest selections yielded three shared biomarkers: SERPINE1, CTHRC1, and EGR1 (**Figure 4G**).

**Figure 4:**
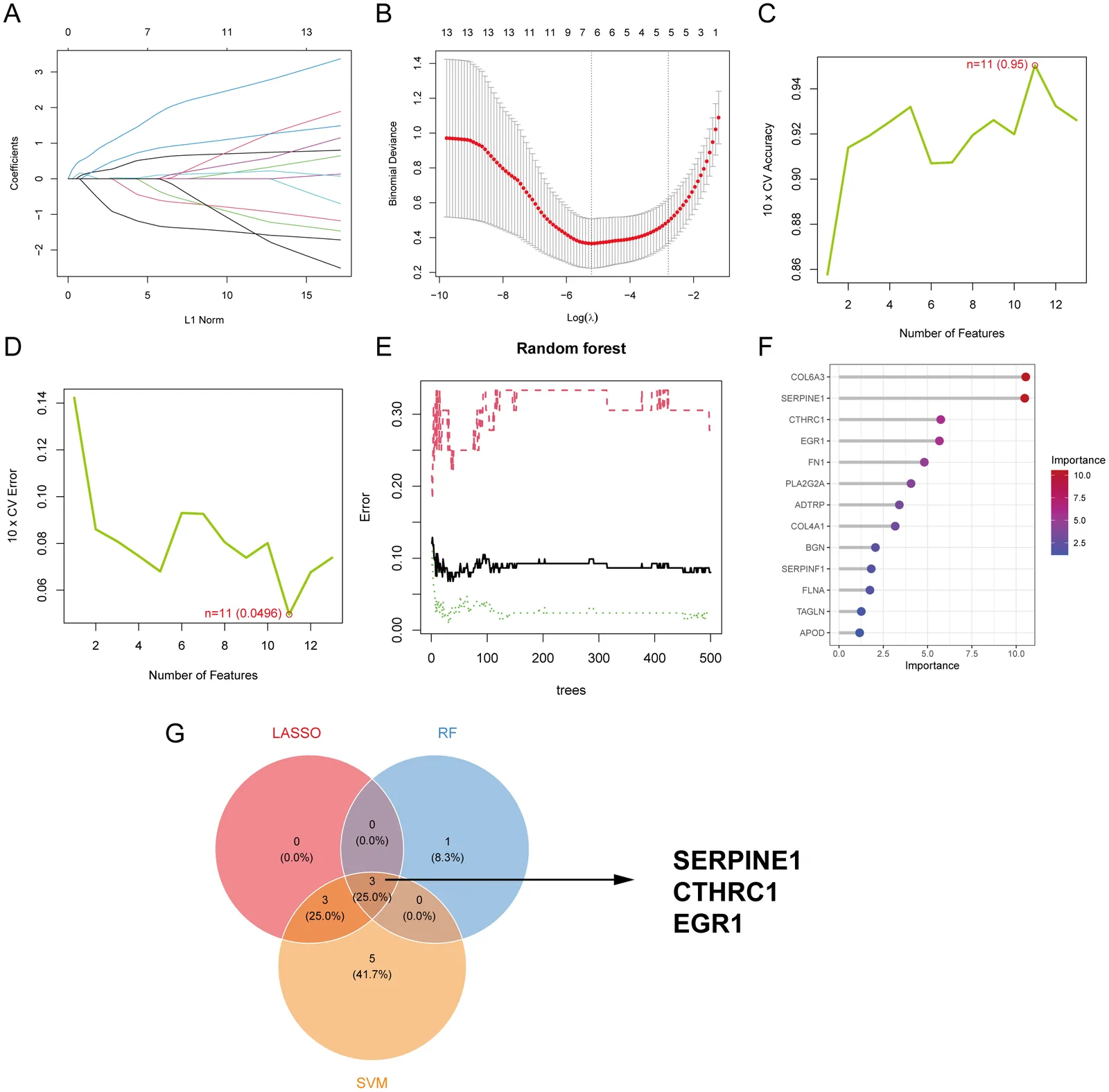
Machine-learning screening identifies SERPINE1, CTHRC1, and EGR1 as key PRG-associated biomarkers. **(A, B)** LASSO coefficient trajectories and 10-fold cross-validation. **(C, D)** SVM-RFE accuracy and error across feature numbers; the optimal subset contained 11 features (accuracy = 0.95; error = 0.0496). **(E, F)** Random-forest error curves across 500 trees and variable-importance ranking. **(G)** Intersection of biomarkers selected by the three algorithms.

The three-gene logistic model showed acceptable calibration and positive decision-curve net benefit across a clinically relevant range of thresholds (**Supplementary Figures 4A,B, E**). In TCGA-STAD, the individual AUCs were 0.912 for SERPINE1, 0.920 for CTHRC1, and 0.878 for EGR1, whereas the integrated model achieved an AUC of 0.935 (**Supplementary Figures 4C, D**). In the platform-specific GEO evaluations, all three genes remained differentially expressed, and the integrated model achieved AUCs of 1.000 in GSE54129 and 0.916 in GSE118916 (**Supplementary Figures 4F–K**). These findings demonstrate consistent diagnostic discrimination across datasets. Because the two GEO datasets also contributed to the upstream discovery workflow, their high AUCs were interpreted as cross-dataset reproducibility rather than completely independent feature-discovery validation.

### 3.3 Mutational burden of the three key genes and associated immune-microenvironment features

Somatic alterations of the three model genes were uncommon in TCGA-STAD. Seventeen tumors contained at least one alteration in EGR1, SERPINE1, or CTHRC1; within this altered subset, EGR1 accounted for 59% of altered cases, SERPINE1 for 35%, and CTHRC1 for 12% (**Supplementary Figure 3A**). Because alterations could co-occur within individual tumors, these percentages are not mutually exclusive. Missense single-nucleotide variants predominated, C>T was the most frequent substitution, and representative variants included SERPINE1 F132Y, CTHRC1 P75L, and EGR1 R322C (**Supplementary Figures 3B–D**).

The altered group showed poorer OS than the unaltered group (P = 0.0446) and poorer disease-free survival (P = 0.0252), whereas the progression-free-survival difference did not reach statistical significance (P = 0.0915; **Supplementary Figure 3E**). TMB was higher in the high-risk group (P = 0.011), and high TMB was associated with inferior survival in this dataset (P = 0.017; **Supplementary Figures 3F,G**). Combined TMB-risk stratification separated four groups with different survival outcomes (P < 0.001; **Supplementary Figure 3H**). The TMB difference between the high-risk and low-risk groups provided a rationale for the subsequent assessment of immune and stromal features in Sections 3.5–3.7. Because the altered subgroup was small and these analyses were not central to the study hypothesis, all mutation and TMB findings are presented in the **Supplementary Information** and are interpreted as exploratory.

### 3.4 Construction, validation, and independent prognostic value of the PRG-associated risk model

The multivariable Cox model generated the following score: risk score = 0.1785 × SERPINE1 + 0.0706 × CTHRC1 + 0.0098 × EGR1. A total of 353 evaluable TCGA-STAD patients were divided into 177 low-risk and 176 high-risk cases. The approximately equal training and testing subsets contained 177 and 176 patients, respectively. Risk-score distributions showed a continuous increase across patients, and the separation point generated balanced high- and low-risk groups in the total, training, and testing cohorts (**Figure 5A**). Death events became more frequent as the score increased (**Figure 5B**), and the expression heatmaps showed progressive enrichment of SERPINE1, CTHRC1, and EGR1 toward the high-risk end of the distribution (**Figure 5C**).

**Figure 5:**
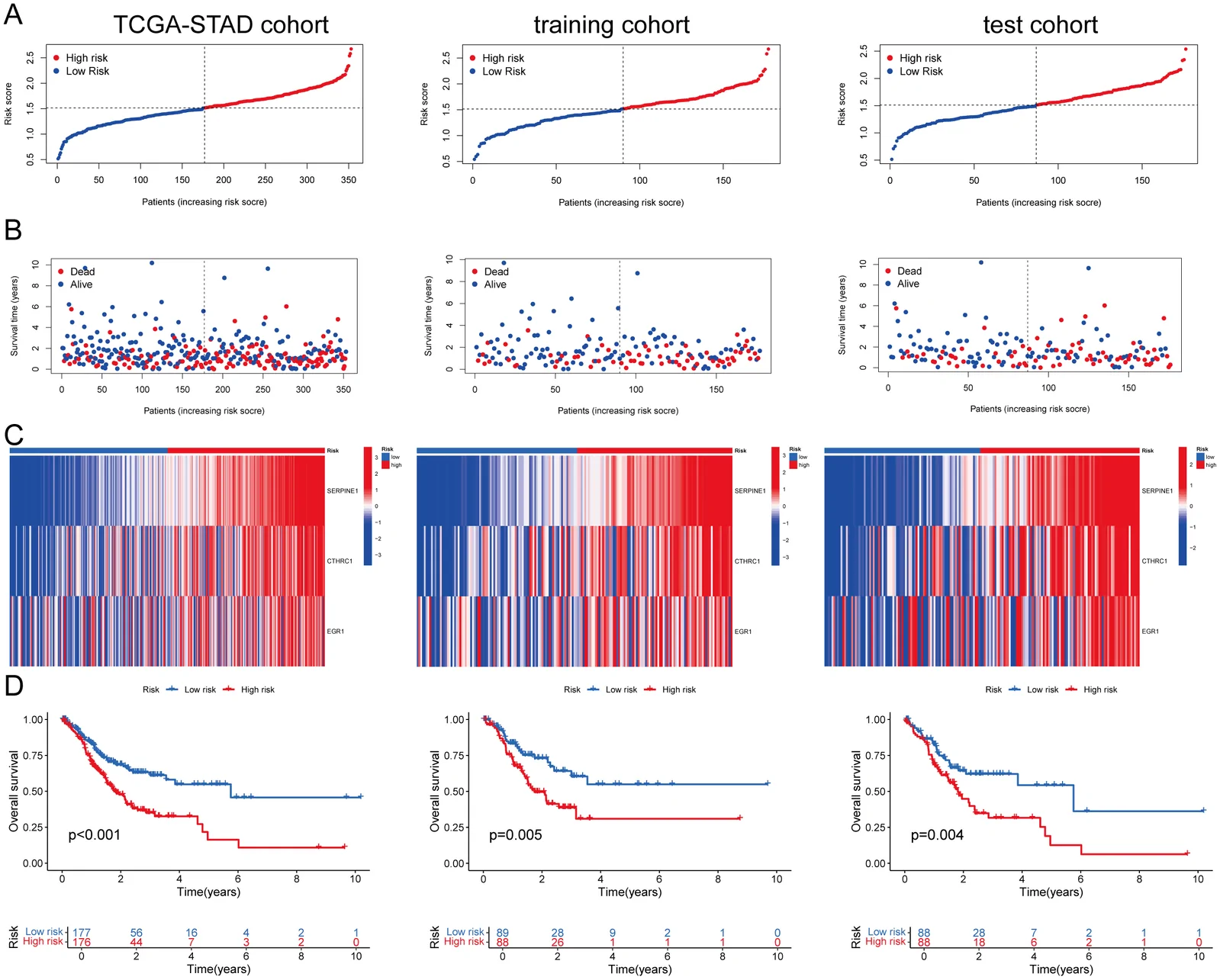
Construction and internal validation of the PRG-associated prognostic model. **(A)** Risk-score distributions in the total TCGA-STAD cohort (n = 353), training cohort (n = 177), and testing cohort (n = 176). **(B)** Survival-status distributions. **(C)** Expression heatmaps for SERPINE1, CTHRC1, and EGR1. **(D)** Kaplan-Meier curves in the total, training, and testing cohorts.

Kaplan-Meier analysis demonstrated significantly poorer OS in the high-risk group in the total TCGA-STAD cohort (P < 0.001), training cohort (P = 0.005), and testing cohort (P = 0.004; **Figure 5D**). The concordant direction in the two internal subsets indicated that the score was not dependent on a single random split. At later follow-up times, the number at risk decreased substantially, particularly in the high-risk group; therefore, the tail of each survival curve was interpreted cautiously.

External evaluation was performed without re-estimating the model coefficients. In GSE84437, high-risk patients had poorer OS (HR = 1.65, 95% CI 1.18-2.30, P = 0.003), with 1-, 3-, and 5-year AUCs of 0.605, 0.669, and 0.679, respectively (**Supplementary Figure 5**). In GSE236522, the high-risk group also had poorer OS (HR = 5.48, 95% CI 1.06-28.30, P = 0.042), and the corresponding AUCs were 0.863, 0.940, and 0.940. The latter cohort included only 33 evaluable cases, as reflected by the wide confidence interval and small numbers at risk; its high AUCs therefore provide supportive but not definitive evidence.

In univariable Cox analysis, age (HR = 1.021, P = 0.018), pathologic stage (HR = 1.900, P < 0.001), T stage (HR = 1.576, P = 0.039), N stage (HR = 1.868, P = 0.004), M stage (HR = 1.688, P = 0.044), and the risk score (HR = 2.759, P < 0.001) were associated with OS (**Figure 6A**). In the multivariable model, age (HR = 1.026, 95% CI 1.008-1.044, P = 0.004), M stage (HR = 1.924, 95% CI 1.142-3.244, P = 0.014), and the risk score (HR = 2.564, 95% CI 1.554-4.230, P < 0.001) remained independently associated with outcome (**Figure 6B**).

**Figure 6:**
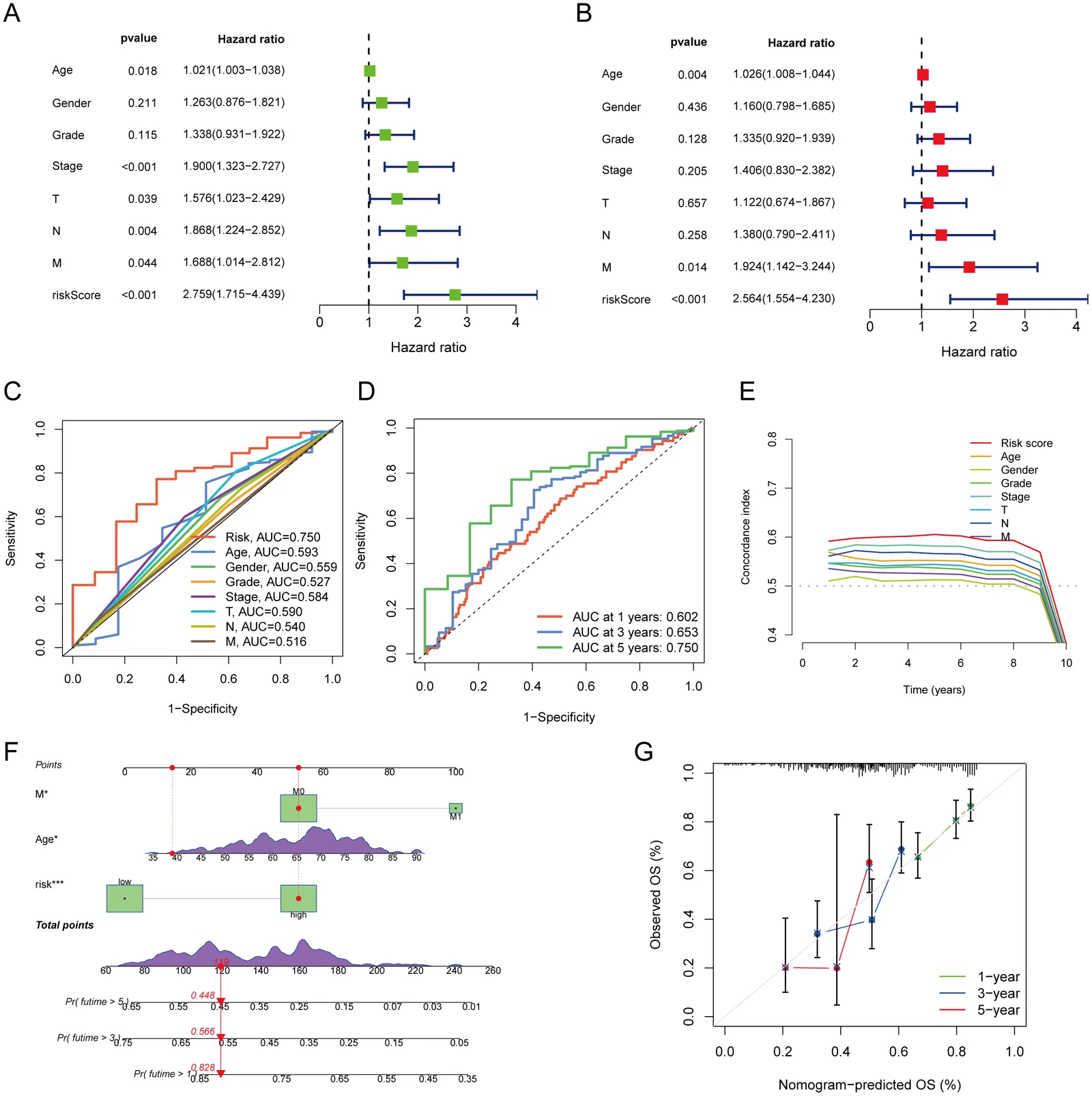
Independent prognostic analysis and nomogram. **(A)** Univariate Cox regression. **(B)** Multivariable Cox regression. **(C,D)** ROC analyses. **(E)** Time-dependent concordance analysis. **(F)** Nomogram. **(G)** Calibration curves.

The risk score achieved an AUC of 0.750 for OS discrimination, compared with 0.593 for age, 0.559 for sex, 0.527 for grade, 0.584 for stage, 0.590 for T stage, 0.540 for N stage, and 0.516 for M stage (**Figure 6C**). Its time-dependent AUC increased from 0.602 at 1 year to 0.653 at 3 years and 0.750 at 5 years (**Figure 6D**), and its concordance index was generally higher than that of the individual clinicopathological variables during most of the observable follow-up period (**Figure 6E**). A nomogram integrating age, M stage, and risk group estimated 1-, 3-, and 5-year OS (**Figure 6F**), and the calibration plots showed overall agreement between predicted and observed probabilities (**Figure 6G**). Subgroup analyses remained directionally consistent in several clinical strata, although nonsignificant findings in smaller subgroups were not interpreted as evidence of no effect (**Supplementary Figure 6**).

### 3.5 Association of the risk score with immune and stromal remodeling

CIBERSORT demonstrated that the high- and low-risk groups differed across multiple immune-cell populations (**Figure 7A**). Relative to low-risk tumors, high-risk tumors showed lower proportions of memory B cells, resting cluster of differentiation 4 (CD4) memory T cells, activated natural killer cells, and eosinophils and higher proportions of resting natural killer cells, M2 macrophages, activated mast cells, and neutrophils. These shifts indicated that the high-risk state was not characterized by a uniform increase in all immune populations but by a redistribution toward selected stromal and myeloid components.

**Figure 7:**
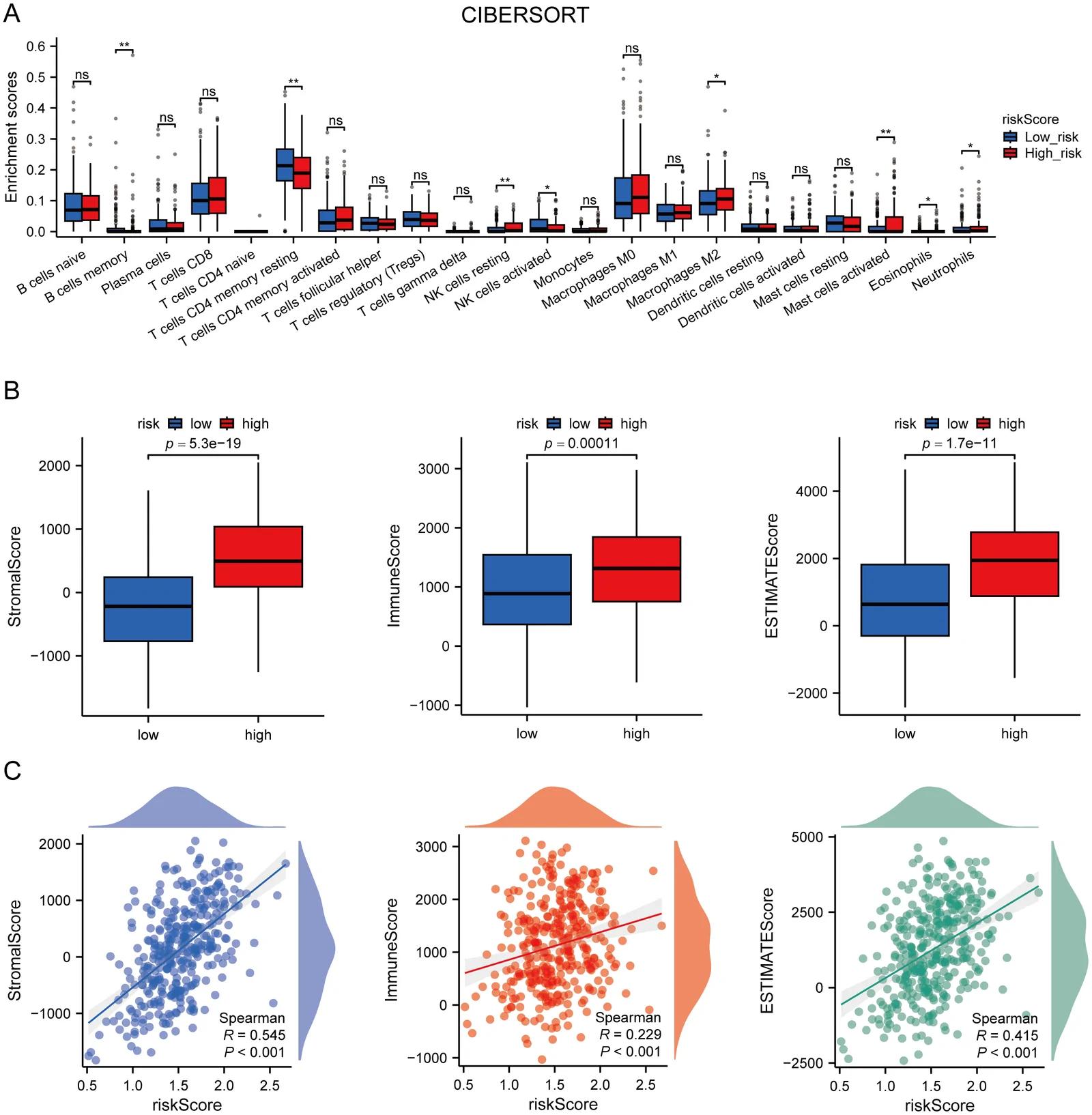
Association between the PRG-associated risk score and the immune microenvironment. **(A)** CIBERSORT comparison between risk groups. **(B)** StromalScore, ImmuneScore, and ESTIMATEScore. **(C)** Spearman correlations between risk score and TME scores.

ESTIMATE analysis provided a consistent tissue-level signal. High-risk tumors had significantly higher StromalScore (P = 5.3 × 10^−19^), ImmuneScore (P = 1.1 × 10^−4^), and ESTIMATEScore (P = 1.7 × 10^−11^; **Figure 7B**). The continuous risk score correlated most strongly with StromalScore (Spearman R = 0.545), followed by ESTIMATEScore (R = 0.415) and ImmuneScore (R = 0.229); all correlations had P < 0.001 (**Figure 7C**). The stronger stromal correlation suggested that extracellular-matrix and fibroblast-associated biology was a major component of the signature.

Complementary analyses using single-sample gene set enrichment analysis (ssGSEA), xCell, TIMER, quanTIseq, Microenvironment Cell Populations-counter (MCP-counter), EPIC, CIBERSORT-ABS, and CIBERSORT showed broadly concordant associations between the risk score and stromal, macrophage, and other immune-cell estimates (**Supplementary Figure 7**).

### 3.6 Single-cell localization of the core PRGs

Single-cell RNA sequencing analysis of STAD_GSE167297 resolved epithelial cells, endothelial cells, fibroblasts, cluster of differentiation 8 (CD8)-positive T cells, B cells, plasma cells, mast cells, dendritic cells, and monocyte/macrophage populations (**Supplementary Figures 8A–C**). The relative abundance of these lineages varied across patients (**Supplementary Figures 8D, E**), emphasizing that the bulk-tissue risk score integrates different cellular compositions rather than reflecting a single malignant-cell population.

SERPINE1 and CTHRC1 expression was concentrated predominantly in fibroblast-related stromal compartments, whereas EGR1 was distributed more broadly across epithelial, stromal, and immune-cell populations (**Supplementary Figures 8F, G**). Correlation analyses showed positive relationships between the model genes and canonical CAF-associated markers, including PDGFRA, PDGFRB, FAP, COL1A1, and extracellular-matrix genes, as well as TAM-associated markers such as CD163, MRC1, MSR1, IL10, and TGFB1 (**Supplementary Figures 8H, I**). These observations indicate that bulk SERPINE1 and CTHRC1 expression partly captures fibroblast abundance or activation. They do not exclude biologically active CTHRC1 expression in malignant epithelial cells, but they show that fibroblasts are a major tissue-level source that must be considered when interpreting the cell-line experiments.

### 3.7 Exploratory immunotherapy-related analyses

High-risk tumors expressed higher levels of multiple checkpoint-related genes, including CD274, CTLA4, HAVCR2, LAG3, PDCD1, PDCD1LG2, and TIGIT, and the continuous risk score was positively correlated with several of these transcripts (**Supplementary Figures 9A–C**). Despite the higher checkpoint expression, the TIDE-predicted responder proportion was lower in the high-risk group (22.15%) than in the low-risk group (49.15%), and high-risk tumors had higher TIDE scores (**Supplementary Figures 9D, E**). Patients with higher TIDE scores also had poorer OS in the analyzed dataset (**Supplementary Figure 9F**). This combination is compatible with an immune-infiltrated but dysfunctional or excluded microenvironment rather than an effectively inflamed antitumor state.

In IMvigor210, patients with complete or partial response had lower risk scores than patients with stable or progressive disease, but the response-prediction AUC was only 0.601, indicating limited cross-cancer discrimination (**Supplementary Figures 9G, H**). High-risk patients also had poorer survival in this urothelial-cancer cohort (HR = 1.36, P = 0.021; **Supplementary Figure 9I**). Because IMvigor210 is not a GC cohort, these findings were treated only as exploratory evidence of transportability.

The institutional GC immunotherapy cohort contained 26 patients with stable/progressive disease and 10 with complete/partial response. Nonresponders showed higher SERPINE1, CTHRC1, and EGR1 expression and a higher composite risk score (**Supplementary Figures 9J, K**). The risk score achieved an AUC of 0.869, compared with 0.754 for SERPINE1, 0.702 for CTHRC1, and 0.806 for EGR1 (**Supplementary Figure 9L**). The small cohort and retrospective response assessment preclude clinical implementation, but the results support prospective evaluation in a larger GC-specific immunotherapy population.

### 3.8 Exploratory GDSC-based drug sensitivity analysis

GC cell lines with matched expression and GDSC response data were ordered by the three-gene risk score and divided at the median (**Supplementary Figure 10A**). The heatmap showed that the high- and low-risk cell-line groups had distinct relative drug-response profiles across the selected agents (**Supplementary Figure 10B**).

High-risk cell lines had significantly higher ln(IC50) values for 5-fluorouracil and trametinib, suggesting lower *in vitro* sensitivity to these agents. Paclitaxel showed a similar directional trend but did not differ significantly. Conversely, the high-risk group had significantly lower ln(IC50) values for cisplatin, oxaliplatin, docetaxel, and gemcitabine, suggesting greater *in vitro* sensitivity. Irinotecan did not differ significantly between groups (**Supplementary Figures 10C–J**).

These results indicate that the three-gene state is associated with pharmacogenomic variation in GC cell lines.

### 3.9 Clinical validation of the three-gene signature

Paired qRT-PCR analysis of 80 GC tissues and their matched adjacent non-tumor tissues showed significantly higher SERPINE1, CTHRC1, and EGR1 mRNA expression in tumors (all P < 0.001; **Figures 8A–C**). The paired design reduced interpatient variability and confirmed that the direction of expression change observed in public datasets was reproduced in the institutional cohort.

**Figure 8:**
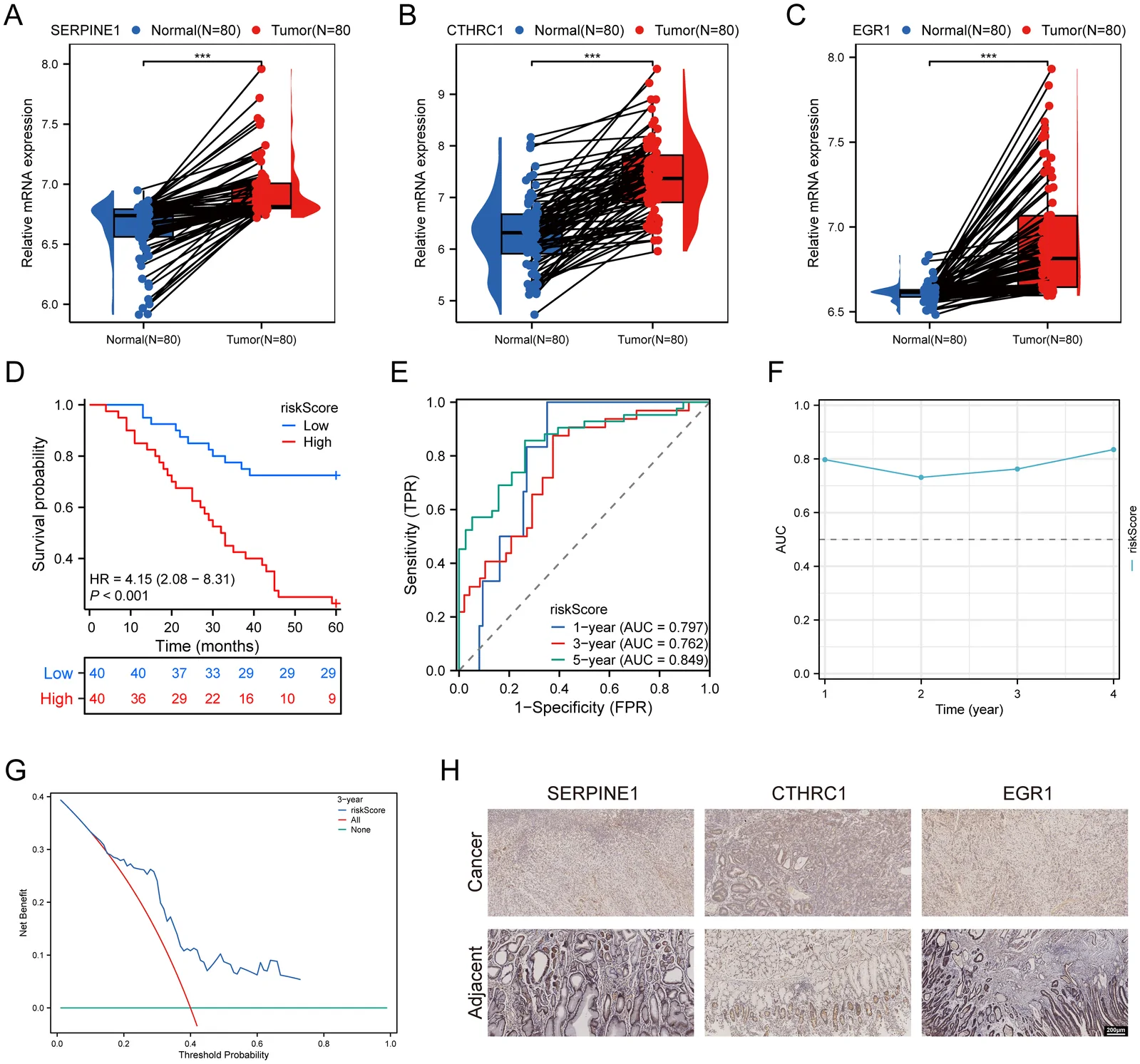
Clinical validation of the three-gene signature. **(A–C)** Paired-tissue quantitative reverse-transcription polymerase chain reaction (qRT-PCR) for SERPINE1, CTHRC1, and EGR1 in 80 gastric cancer (GC) tissues and matched adjacent non-tumor tissues; paired Wilcoxon signed-rank tests were used. **(D)** Kaplan–Meier analysis in the 80-patient cohort, with hazard ratio (HR) and 95% confidence interval (CI). **(E)** Time-dependent receiver operating characteristic (ROC) curves for 1-, 3-, and 5-year overall survival (OS). **(F)** Longitudinal time-dependent area under the curve (AUC). **(G)** Three-year decision-curve analysis. **(H)** Representative immunohistochemical staining of SERPINE1, CTHRC1, and EGR1 in GC and adjacent tissues; scale bar, 200 µm. Representative staining was not used as a substitute for quantitative histochemical-score (H-score) analysis. ***P < 0.001.

The clinical risk score divided the 80 patients into 40 high-risk and 40 low-risk cases. High-risk patients had markedly poorer OS (HR = 4.15, 95% CI 2.08-8.31, P < 0.001; **Figure 8D**). The time-dependent AUCs were 0.797 at 1 year, 0.762 at 3 years, and 0.849 at 5 years (**Figure 8E**), and the longitudinal AUC remained above the no-discrimination threshold throughout the evaluated follow-up (**Figure 8F**). Three-year decision-curve analysis showed a positive net benefit for the risk score across a range of threshold probabilities compared with the treat-all and treat-none strategies (**Figure 8G**).

Representative immunohistochemical images for all three model genes are shown in **Figure 8H**. CTHRC1 showed the clearest tumor-associated protein staining pattern. SERPINE1 and EGR1 displayed heterogeneous staining between epithelial and stromal regions and were not accompanied by formal histochemical scores (H-scores) in the present dataset. Accordingly, the representative images demonstrate that all three proteins were evaluated but were not used to claim uniform protein-level upregulation for SERPINE1 and EGR1.

### 3.10 CTHRC1 knockdown suppresses malignant phenotypes *in vitro*

Baseline qRT-PCR showed that SERPINE1, CTHRC1, and EGR1 were expressed at higher levels in AGS, HGC-27, and MKN-45 GC cells than in GES-1 cells (all P < 0.001; **Figures 9A–C**). Three independent CTHRC1-targeting shRNAs were screened in HGC-27 and AGS cells. All reduced CTHRC1 mRNA, and sh-CTHRC1–3 produced the most consistent reduction across both cell lines; Western blotting confirmed the corresponding decrease in CTHRC1 protein (**Figures 9D, E**). This construct was therefore used for subsequent functional experiments.

**Figure 9:**
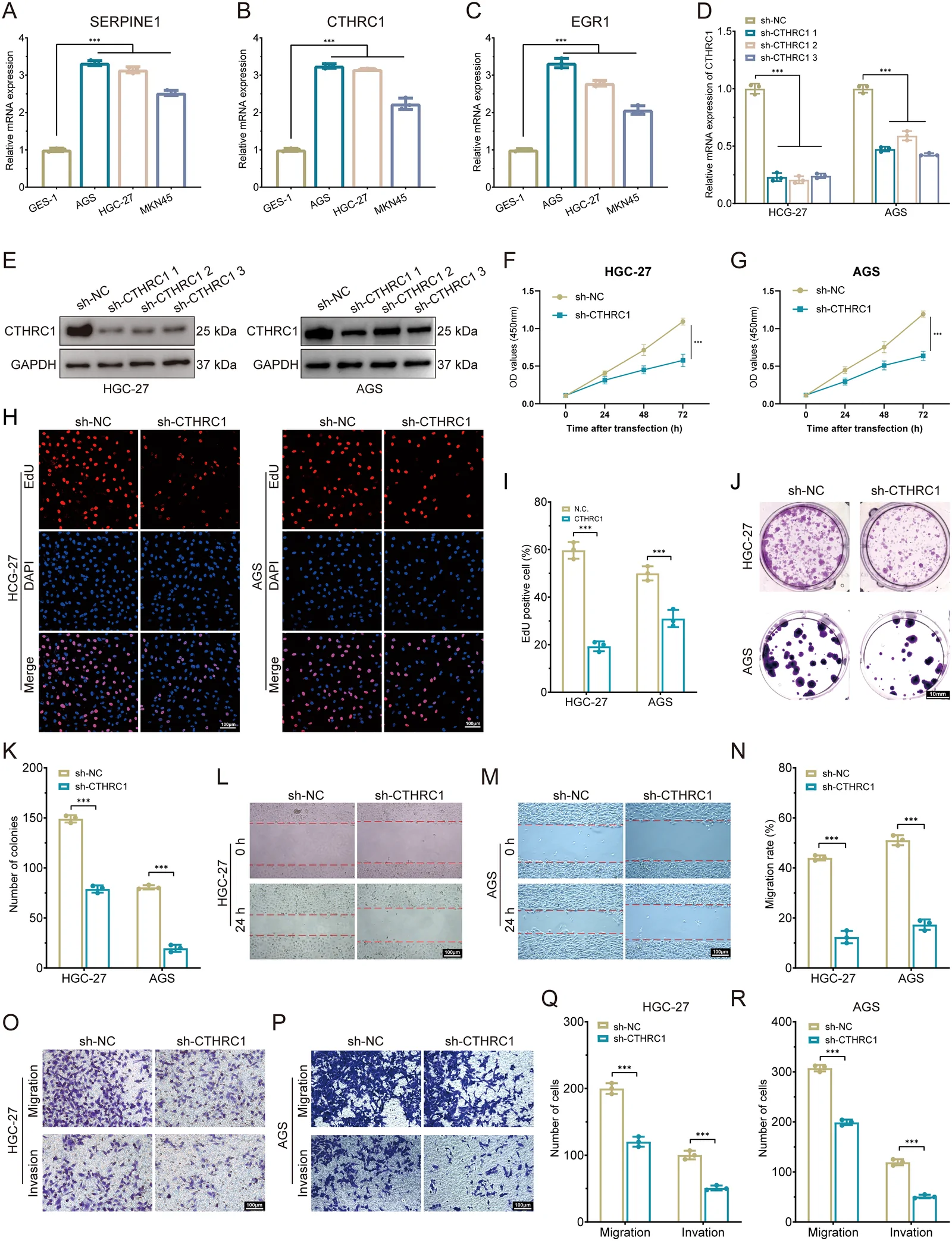
Cellular expression and functional validation of CTHRC1 *in vitro*. **(A–C)** Quantitative reverse-transcription polymerase chain reaction (qRT-PCR) analysis of SERPINE1, CTHRC1, and EGR1 in GES-1, AGS, HGC-27, and MKN-45 cells. **(D)** qRT-PCR screening of three CTHRC1-targeting short hairpin RNAs (shRNAs) in HGC-27 and AGS cells. **(E)** Western blot validation of CTHRC1 knockdown; glyceraldehyde-3-phosphate dehydrogenase (GAPDH) was the loading control. **(F, G)** Cell Counting Kit-8 (CCK-8) proliferation assays. **(H, I)** Representative 5-ethynyl-2′-deoxyuridine (EdU) staining and quantification; scale bar, 100 µm. **(J, K)** Colony-formation assays and quantification; scale bar, 10 mm. **(L–N)** Wound-healing assays and migration-rate quantification; scale bar, 100 µm. **(O–R)** Transwell migration/invasion assays and quantification; scale bar, 100 µm. Data are mean ± standard deviation (SD) from three independent biological experiments. **(A–D)** were analyzed by one-way analysis of variance (ANOVA) with Dunnett correction, panels F and G by two-way ANOVA with multiple-comparison correction, and two-group endpoint comparisons by two-sided Student’s t-tests. ***P < 0.001.

CTHRC1 knockdown reduced the increase in CCK-8 absorbance over 72 h in both HGC-27 and AGS cells (**Figures 9F, G**). EdU imaging showed fewer DNA-synthesizing cells after knockdown, and quantification demonstrated an approximately one-half reduction in EdU-positive fractions in both cell lines (**Figures 9H, I**). Colony formation was also markedly impaired, with fewer and smaller colonies in the sh-CTHRC1 groups (**Figures 9J, K**). All quantitative comparisons were significant at P < 0.001.

Cell motility and invasive behavior were similarly suppressed. At 24 h, wound closure was substantially lower after CTHRC1 knockdown in HGC-27 and AGS cells (**Figures 9L–N**). Transwell assays showed fewer migrated cells in the uncoated inserts and fewer invaded cells through Matrigel-coated membranes (**Figures 9O–R**). The reductions in both migration and invasion were significant in each cell line (P < 0.001).

Together, these experiments demonstrate that cancer-cell-intrinsic CTHRC1 supports proliferation, DNA synthesis, clonogenic survival, migration, and invasion in the tested GC cell lines.

### 3.11 CTHRC1 knockdown suppresses xenograft growth and increases apoptosis

Subcutaneous tumors generated from sh-CTHRC1 cells were visibly smaller than sh-NC tumors at the experimental endpoint (**Figure 10A**). Mean tumor weight was markedly reduced in the sh-CTHRC1 group, and serial measurements showed progressively slower tumor growth, with the difference becoming pronounced during the later observation period (both P < 0.001; **Figures 10B, C**). Five mice were analyzed in each group.

**Figure 10:**
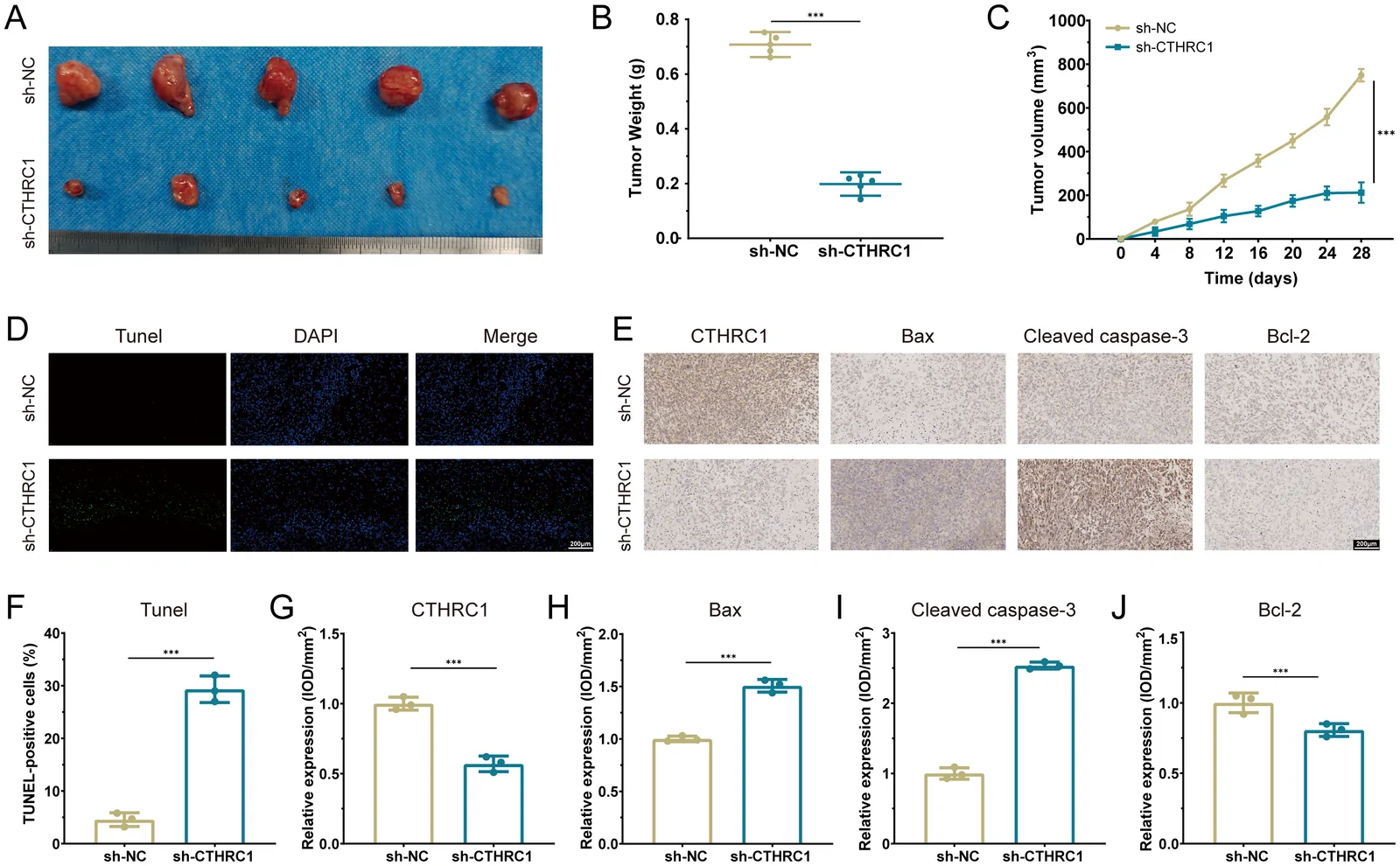
CTHRC1 knockdown inhibits gastric cancer (GC) xenograft growth and promotes apoptosis. **(A)** Representative excised tumors. **(B)** Tumor weight. **(C)** Tumor-growth curve. **(D, F)** Terminal deoxynucleotidyl transferase dUTP nick-end labeling (TUNEL) staining and quantification. **(E,G–J)** Immunohistochemistry and quantification of CTHRC1, Bax, cleaved caspase-3, and Bcl-2. Tumor growth and weight: n = 5 mice per group; histological quantification: three independent tumors per group. Tumor-growth curves were analyzed by two-way analysis of variance (ANOVA); endpoint and histological comparisons used two-sided Student’s t-tests. ***P < 0.001.

TUNEL staining showed a substantial increase in apoptotic cells after CTHRC1 knockdown (**Figures 10D, F**). Immunohistochemistry confirmed lower CTHRC1 expression in the knockdown tumors and demonstrated an apoptosis-associated shift: Bax and cleaved caspase-3 staining increased, whereas Bcl-2 staining decreased (all P < 0.001; **Figures 10E, G–J**). These findings were consistent with the reduced tumor growth and indicated that CTHRC1 supports an anti-apoptotic phenotype *in vivo*.

## 4 Discussion

This study developed a three-gene transcriptional signature derived from a GeneCards-defined palmitoylation-associated gene set and evaluated its diagnostic, prognostic, immune-stromal, and pharmacogenomic associations in GC. The principal strengths are the integration of public discovery datasets, internal and external prognostic evaluation, an 80-pair institutional tissue cohort, single-cell localization, and functional validation of CTHRC1 *in vitro* and *in vivo*. The score consistently identified patients with poorer survival, and the strongest biological feature of the high-risk group was a stroma-rich, immune-remodeled microenvironment. Nevertheless, the study does not demonstrate that the score is a direct measure of global palmitoylation or that the observed CTHRC1 phenotype is mediated by a palmitoylation-dependent mechanism.

The palmitoylation framework therefore requires biological restraint. S-acylation can regulate protein trafficking, membrane residence, signaling, immune escape, and cell-death susceptibility (10–13). Recent mechanistic studies further show that ZDHHC8-mediated palmitoylation of glutathione peroxidase 4 (GPX4) can alter ferroptosis sensitivity and antitumor immunity, that palmitoylation of programmed death-ligand 1 (PD-L1) stabilizes this checkpoint protein, and that DHHC20-mediated palmitoylation of epidermal growth factor receptor (EGFR) reshapes oncogenic signaling dependence (48–50). These studies establish that palmitoylation is relevant to cancer biology, but they do not imply that expression of every gene returned by a GeneCards search represents cellular palmitoylation activity. In the present work, PRG status is a pathway-oriented annotation used for transcriptomic screening. Direct acyl-biotin-exchange, acyl-resin-assisted-capture, metabolic labeling, site-specific mutagenesis, or palmitoyl-proteomic experiments are required to connect the signature to specific lipid-modification events.

Several GC prognostic models have been derived from tumor-microenvironment, cancer-associated fibroblast (CAF), immune, lipid-homeostasis, and other pathway-defined gene sets (51–57). These studies demonstrate that transcriptomic scores can capture clinically relevant heterogeneity, but most were developed mainly from retrospective public datasets and did not combine external prognostic evaluation, paired institutional tissues, cell-type localization, and experimental perturbation of a model gene. The principal strength of the present study is therefore the integrated evidence chain rather than a claim of numerical superiority: a compact three-gene score was evaluated in internal and external cohorts, reproduced in an 80-patient institutional cohort, localized at single-cell resolution, and linked to *in vitro* and *in vivo* CTHRC1 phenotypes. Nevertheless, the moderate 1- and 3-year discrimination in some cohorts, the small GSE236522 cohort, and the absence of head-to-head recalculation against competing signatures mean that this model should be regarded as complementary to existing GC classifiers. Similar integrative signature-building strategies have been applied across other tumor types to link pathway-defined gene programs with prognosis and immunotherapy-related features, including ubiquitination-, m7G-, disulfidptosis-, migrasome-, cuproptosis-, and anoikis-related models (58–65), with recent multi-omics studies further combining computational discovery with experimental or clinical validation (66, 67).

Recent high-resolution studies provide direct biological context for our results. Single-cell analyses have documented extensive reprogramming of stromal, myeloid, dendritic, and exhausted T-cell states in GC (68), identified a pro-invasive CAF subgroup associated with unfavorable clinical outcomes (69), and mapped more than 200,000 cells across clinical stages and histologic subtypes (70). Independent work further demonstrated active communication among malignant, stromal, and immune-cell populations (71) and showed that immune and stromal ecotypes evolve during gastric adenocarcinoma progression (72). Together, these studies support our interpretation that the three-gene score reflects a multicellular tumor state rather than a purely tumor-cell-intrinsic program.

The strongest signal in the present study was stromal activation. High-risk tumors had markedly higher StromalScore and ESTIMATEScore, and the risk score correlated more strongly with the stromal component than with the immune component. This pattern is consistent with reports that immunosuppressive MFAP2-positive CAFs are associated with poor prognosis and therapeutic resistance in GC (73), that extracellular-matrix composition and fibroblast programs vary across gastric histologic subtypes (74), and that spatially defined CAF subsets participate in extensive ligand-receptor communication with malignant and immune cells (75). A recent spatial study further identified immunosuppressive crosstalk between CCL2-positive fibroblasts and STAT3-activated macrophages in GC (76). These observations strengthen the conclusion that the high-risk state represents coordinated fibroblast-ECM-myeloid remodeling, not a uniform increase in immune infiltration.

Single-cell localization is critical to this interpretation. SERPINE1 and CTHRC1 were predominantly enriched in fibroblast-related compartments and correlated with CAF and tumor-associated macrophage markers. This finding agrees with previous GC analyses that placed CTHRC1 mainly in fibroblast clusters (77) and with histological evidence that CTHRC1 can be produced predominantly by activated stromal cells in several human solid tumors, including gastric cancer (78). Thus, bulk-tissue expression may reflect stromal abundance and activation as well as expression within malignant epithelial cells. This explains why a high tissue-level CTHRC1 signal can coexist with detectable and biologically active CTHRC1 in GC cell lines. Spatial transcriptomics, dual immunofluorescence, ligand-receptor analysis, conditioned-medium experiments, and tumor-cell/CAF co-culture will be required to quantify the relative contribution of each compartment.

The functional results show that reducing CTHRC1 in AGS and HGC-27 cells suppresses proliferation, DNA synthesis, clonogenicity, migration, and invasion *in vitro*; CTHRC1 knockdown also suppresses xenograft growth and increases apoptosis *in vivo*. These results are consistent with previous reports linking CTHRC1 to poor prognosis and immune escape (79), HIF-1α/CXCR4-mediated metastasis (80), and angiogenic or macrophage-related microenvironmental programs in GC (77). The present study adds direct loss-of-function evidence in two GC cell lines and a xenograft model, but it does not establish that CTHRC1 is a palmitoylation-dependent driver. The observed phenotypes may involve Wnt/planar-cell-polarity, transforming growth factor beta/extracellular-matrix, hypoxia, PI3K-Akt, or focal-adhesion signaling; rescue experiments using shRNA-resistant CTHRC1, measurement of secreted CTHRC1, pathway-specific inhibitors, and phosphorylation analyses are required to define the downstream mechanism.

The immunotherapy analyses should also be interpreted as associative. A prospective metastatic-GC study showed that response to PD-1 blockade is influenced by established tumor features such as microsatellite instability, Epstein-Barr virus status, PD-L1 expression, and dynamic circulating tumor DNA (81). Our risk score should therefore be viewed as a potential microenvironmental complement to, rather than a replacement for, clinically established biomarkers. More broadly, tumor progression and immunotherapy response are shaped by the tumor microenvironment, macrophage-mediated immune regulation, the cancer-immunity cycle and immune checkpoints, pan-cancer immune states, and tumor mutational burden (82–91). The combination of higher checkpoint expression, higher TIDE scores, and a lower predicted responder proportion in the high-risk group is compatible with an immune-infiltrated but dysfunctional or excluded state. This interpretation is also supported by the spatially resolved CCL2-positive fibroblast/STAT3-activated macrophage axis described in GC (76) and by broader evidence that TGF-β-rich stroma can restrict T-cell access (92). Because IMvigor210 is a urothelial-cancer cohort and the institutional GC immunotherapy cohort was small and retrospective, prospective GC cohorts receiving standardized checkpoint blockade are required before treatment-selection claims can be made.

The GDSC and DGIdb analyses are similarly hypothesis-generating. GDSC indicated that high-risk cell lines were less sensitive *in vitro* to 5-fluorouracil and trametinib and more sensitive to several platinum, taxane, and gemcitabine treatments. These differences do not establish patient-level treatment response because cell-line pharmacogenomic resources do not reproduce pharmacokinetics, combination therapy, immune effects, intratumoral heterogeneity, or stromal protection (20). DGIdb edges can represent diverse interaction types and do not prove direct inhibition of SERPINE1, CTHRC1, or EGR1 or efficacy in GC (44). Candidate compounds should be prioritized according to interaction type and existing pharmacology and then tested in GC cell lines, patient-derived organoids, CAF co-cultures, and *in vivo* models.

Several limitations remain. The analyses were retrospective and subject to platform differences and incomplete clinical annotation. Although the two discovery GEO datasets were normalized separately and adjusted with ComBat after merging, cross-platform analyses still used dataset-specific expression scales. External prognostic performance varied, and GSE236522 and the institutional immunotherapy cohort were small. The model was not benchmarked against competing published signatures in identical samples. Representative IHC images were provided for all three genes, but quantitative protein-level validation and functional experiments were concentrated on CTHRC1; the heterogeneous SERPINE1 and EGR1 staining patterns require standardized H-score analysis. The immunodeficient subcutaneous xenograft model confirms a tumor-cell-autonomous phenotype but cannot evaluate immune exclusion, fibroblast-derived CTHRC1, or stromal interactions; orthotopic, humanized, or stromal co-implantation models will be required. Most importantly, no direct palmitoylation assay was conducted. Multicenter prospective validation, direct palmitoylation measurements, spatial analysis, CAF-tumor co-culture, and mechanistic rescue studies are required before clinical or mechanistic claims can be strengthened.

In summary, the SERPINE1/CTHRC1/EGR1 score identifies a GC subgroup characterized by poorer survival and prominent immune-stromal remodeling and provides exploratory pharmacogenomic information. CTHRC1 has a tumor-promoting role in the tested cell and xenograft models, whereas its predominant fibroblast localization in patient single-cell data indicates that stromal and epithelial sources must both be considered. The findings support a palmitoylation-associated transcriptional framework, not a proven palmitoylation-dependent mechanism.

## 5 Conclusion

This study identifies SERPINE1, CTHRC1, and EGR1 as a compact palmitoylation-associated transcriptional signature with prognostic and immune-stromal relevance in GC. The score was evaluated in internal, external, and institutional cohorts and was associated with exploratory immunotherapy- and drug-sensitivity-related features. Functional experiments support a tumor-promoting role for CTHRC1 in the tested GC models. Direct palmitoylation assays and prospective clinical validation are required before the signature can be considered a palmitoylation-dependent biomarker or a treatment-selection tool.

## Data Availability

The datasets presented in this study can be found in online repositories. The names of the repository/repositories and accession number(s) can be found in the article/**Supplementary Material**.
