# Supplementary material for: A palmitoylation-associated three-gene signature predicts prognosis, immune features, and drug sensitivity in gastric cancer: functional validation of CTHRC1
Source: Front Immunol. 2026 Sep 25;17:1893438. doi: 10.3389/fimmu.2026.1893438 (PMC13649694; doi:10.3389/fimmu.2026.1893438)
Supplement: Supplementary file 1 [file DataSheet1.zip › Supplementary Figures.docx]

**Supplementary Figures**

**Figure S1.** WGCNA of GC-associated gene modules. (A) Soft-thresholding power analysis. (B) Module–trait relationship heatmap. (C) Gene dendrogram and module colors. (D) Module membership versus gene significance in the blue module. This figure was moved from the Supplementary Information to the main manuscript.

**Figure S2.** Immune infiltration analysis associated with DEPRGs. (A) ssGSEA-based immune infiltration heatmap. (B) Correlation heatmap between DEPRGs and immune cell subsets. (C) Violin plots showing immune infiltration differences between gastric cancer and control tissues.

**Figure S3.** Somatic mutation features and TMB-related prognostic analysis of core PRGs. (A) Oncoplot of SERPINE1, CTHRC1 and EGR1 mutations. (B,C) Variant summaries and SNV classes. (D) Lollipop plots of core PRGs. (E) Survival analysis of altered and unaltered groups. (F) TMB distribution between risk groups. (G,H) Survival analyses based on TMB and combined TMB/risk stratification.

**Figure S4.** Diagnostic performance of the three-gene PRG model. (A) Diagnostic nomogram. (B) Calibration curve. (C,D) ROC curves in the training cohort. (E) Decision curve analysis. (F-K) Validation in GSE54129 and GSE118916.

**Figure S5.** External validation of the PRG risk model in independent GEO cohorts. (A) Risk score distributions, survival status plots and expression heatmaps in GSE84437 and GSE236522. (B) Kaplan-Meier curves for OS in GSE84437 and GSE236522. (C) Time-dependent ROC curves for 1-, 3- and 5-year OS prediction in GSE84437 and GSE236522.

**Figure S6.** Clinicopathological subgroup analysis of the PRG risk score. (A) Heatmap showing distributions of clinical characteristics and risk groups. (B) Kaplan-Meier curves comparing high- and low-risk groups across clinicopathological strata.

**Figure S7.** Immune infiltration patterns associated with PRG expression and risk groups. (A) CIBERSORT-derived immune cell composition according to the expression levels of SERPINE1, CTHRC1, and EGR1. (B) ssGSEA-based comparison of immune cell enrichment between low- and high-risk groups. (C) Correlations between the PRG risk score and immune infiltration estimates derived from multiple algorithms.

**Figure S8.** Single-cell RNA-seq analysis of core PRGs. (A,B) UMAP clustering and cell annotation. (C) Marker dot plot. (D,E) Cell composition. (F,G) SERPINE1, CTHRC1 and EGR1 expression across cell types. (H,I) Correlations with CAF and TAM markers.

**Figure S9.** Association between PRG risk score and immunotherapy response. (A-C) Immune checkpoint analyses. (D,E) Predicted response and TIDE score. (F) Survival according to TIDE. (G-I) IMvigor210 exploratory validation. (J-L) Clinical gastric cancer validation.

**Figure S10.** GDSC-based drug sensitivity analysis associated with the PRG risk score. (A) Distribution of PRG risk scores in gastric cancer cell lines. Cell lines were ordered by increasing risk score and divided into high- and low-risk groups according to the median cutoff. (B) Heatmap showing relative ln(IC50)-based drug response patterns between high- and low-risk groups. (C-J) Comparison of ln(IC50) values for representative chemotherapeutic and targeted agents between high- and low-risk groups, including 5-fluorouracil, cisplatin, oxaliplatin, paclitaxel, docetaxel, irinotecan, gemcitabine and trametinib. Lower ln(IC50) values indicate greater drug sensitivity. ns, not significant; **P < 0.01; ***P < 0.001.
